# Bit-parallel sequence-to-graph alignment

**DOI:** 10.1093/bioinformatics/btz162

**Published:** 2019-03-09

**Authors:** Mikko Rautiainen, Veli Mäkinen, Tobias Marschall

**Affiliations:** 1 Center for Bioinformatics, Saarland University, Saarland Informatics Campus E2.1, 66123 Saarbrücken, Germany; 2 Max Planck Institute for Informatics, Saarland Informatics Campus E1.4, 66123 Saarbrücken, Germany; 3 Saarbrücken Graduate School of Computer Science, Saarland University, Saarland Informatics Campus E1.3, 66123 Saarbrücken, Germany; 4 Department of Computer Science, University of Helsinki, Helsinki, Finland

## Abstract

**Motivation:**

Graphs are commonly used to represent sets of sequences. Either edges or nodes can be labeled by sequences, so that each path in the graph spells a concatenated sequence. Examples include graphs to represent genome assemblies, such as string graphs and de Bruijn graphs, and graphs to represent a pan-genome and hence the genetic variation present in a population. Being able to align sequencing reads to such graphs is a key step for many analyses and its applications include genome assembly, read error correction and variant calling with respect to a variation graph.

**Results:**

We generalize two linear sequence-to-sequence algorithms to graphs: the *Shift-And* algorithm for exact matching and Myers’ *bitvector* algorithm for semi-global alignment. These linear algorithms are both based on processing *w* sequence characters with a constant number of operations, where *w* is the word size of the machine (commonly 64), and achieve a speedup of up to *w* over naive algorithms. For a graph with |V| nodes and |E| edges and a sequence of length *m*, our bitvector-based graph alignment algorithm reaches a worst case runtime of $O\left(|V|+\left\lceil \frac{m}{w}\right\rceil |E|\;\text{log}\;w\right)$ for acyclic graphs and O(|V|+m|E| log w) for arbitrary cyclic graphs. We apply it to five different types of graphs and observe a speedup between 3-fold and 20-fold compared with a previous (asymptotically optimal) alignment algorithm.

**Availability and implementation:**

https://github.com/maickrau/GraphAligner

**Supplementary information:**

Supplementary data are available at *Bioinformatics* online.

## 1 Introduction

Aligning two sequences is a classic problem in bioinformatics. The standard dynamic programming (DP) algorithm, introduced by Needleman and Wunsch (1970), aligns two sequences of length *n* in $O(n^{2})$ time. Countless variants of this classic DP algorithm exist, in particular its generalization to local alignment (Smith and Waterman, 1981), where the alignment can be between any substrings of the two sequences, and semi-global alignment (Sellers, 1980) where one sequence (*query*) is entirely aligned to a substring of the other (*reference*).

Recent projects such as the 1000 Genomes Project (1000 Genomes Project Consortium *et al.*, 2015) have provided genetic variants for many individuals. Currently, we witness a strong interest in *pan-genomic* methods for representing and analyzing the variations between individual genomes in a manner that avoids duplicate work in the shared genomic areas (Computational Pan-Genomics Consortium, 2018; Danek *et al.*, 2014; Rahn *et al.*, 2014). One such method is to use a graph as the reference, which provides a simple way of representing both shared and unique areas, and can represent complex variations as well (Garrison *et al.*, 2018; Paten *et al.*, 2017). In addition to representing genomic diversity, graphs whose nodes or edges are labeled by characters are commonly used in many other applications in bioinformatics, for instance genome assembly (Compeau *et al.*, 2011; Miller *et al.*, 2010) and multiple sequence alignment (Kehr *et al.*, 2014). With an increasing usage of graphs, algorithms for aligning reads to graphs are also of growing interest and have already been applied successfully for purposes such as genome assembly (Antipov *et al.*, 2016) and error correction (Salmela and Rivals, 2014). So far, however, algorithms to align sequences to graphs while exploiting bit-parallelism have been lacking.

In this article, we study the semi-global sequence-to-graph alignment problem. That is, we seek to find a path in a directed, node-labeled graph that has minimum edit distance to the query sequence. We use the edit distance formulation by Levenshtein (1966), with unit costs for mismatches and indels.

*Related work.* Already in 1989, an algorithm for approximate regular expression matching was discovered (Myers and Miller, 1989). It represented the regular expression as a graph and achieved a runtime of O(|V|+m|E|) for aligning a sequence to it, where |V| is the number of nodes, |E| is the number of edges and *m* is the lengths of the query sequence. In 2000, an O(|V|+m|E|) algorithm for aligning a sequence to an arbitrary graph was discovered in the context of hypertext searching (Navarro, 2000). The algorithm is a generalization of the Needleman–Wunsch algorithm. It proceeds row-wise with two sweeps per row: on the first sweep, calculating the recurrence from the values in the previous row, and on the second sweep, propagating the recurrence term for the values in the same row with a depth first search.

Other algorithms for sequence-to-graph alignment have been discovered in the context of bioinformatics; however, although published later than the O(|V|+m|E|) algorithms, they either obtain worse runtimes, do not apply to arbitrary graphs, or do not produce the optimal alignment. We list these results below for completeness. *Partial order alignment* (Lee *et al.*, 2002) (POA) extends standard DP to directed acyclic graphs (DAG) in O(|V|+m|E|) time but does not handle cyclic graphs. The variation graph tool vg (Garrison *et al.*, 2018) aligns to cyclic graphs by ‘unrolling’ the graph into a DAG, and then uses POA. However, unrolling the graph can produce a drastically larger DAG (Vaddadi *et al.*, 2017). V-align (Vaddadi *et al.*, 2017) aligns to arbitrary graphs with O((|V′|+1)m|E|) runtime where |V′| is the size of the graph’s minimum feedback vertex set. Limasset *et al.* (2016) align reads to de Bruijn graphs, but in a heuristic manner without guaranteeing optimal alignment. The genome assembler hybridSPAdes (Antipov *et al.*, 2016) re-phrases sequence-to-graph alignment as a shortest path problem and uses Dijkstra’s algorithm, leading to O(|E|m+|V|m log(|V|m)) runtime. Dilthey *et al.* (2015, 2016) align reads to a *population reference graph*, which does not allow cycles.

*Contributions.* In this article, we introduce techniques for bit-parallel semi-global sequence-to-graph alignment. To illustrate some of the central ideas, we first discuss the simpler question of generalizing the *Shift-And* algorithm (Baeza-Yates and Gonnet, 1992; Dömölki, 1964, 1968) for exact string matching to graphs. We obtain an algorithm with an $O\left(\left|V|+\left\lceil \frac{m}{w}\right\rceil |E\right|\right)$ runtime in acyclic graphs, matching the Shift-And algorithm for linear sequences, and O(|V|+m|E|) runtime in arbitrary cyclic graphs. We then generalize Myers’ *bitvector* alignment algorithm (Myers, 1999) to graphs, which proceeds along the same lines as the Shift-And algorithm, but requires some further algorithmic insights to handle nodes with an in-degree greater than one. We arrive at an algorithm with a runtime of $O\left(|V|+\left\lceil \frac{m}{w}\right\rceil |E|\;\text{log}\;w\right)$ for acyclic graphs and O(|V|+m|E| log w) for arbitrary cyclic graphs. Moreover, we perform experiments showing that despite the higher time complexity in cyclic graphs, the bitvector algorithm is empirically faster than the O(|V|+m|E|) algorithm for hypertext searching (Navarro, 2000) by a factor of 3 to 20, depending on the input graph.

## 2 Problem definition

Definition 1 (Sequence graph). *We define a sequence graph as a tuple*G=(V,E,σ)*, where*$V=\{v_{1},\ldots ,v_{n}\}$*is a finite set of nodes*, E⊂V×V*is a set of directed edges and*σ:V→Σ*assigns one character from the alphabet Σ to each node. We refer to the sets of indices of in-neighbors and out-neighbors of node v_i_ as*$\delta_{i}^{\mathrm{in}}:=\left\{i\prime \in \{1,\ldots ,n\}\;|\;(v_{i\prime },v_{i})\in E\right\}$*and*$\delta_{i}^{\text{out}}:=\left\{i\prime \in \{1,\ldots ,n\}\;|\;(v_{i},v_{i\prime })\in E\right\}$*, respectively*.

Definition 2 (Path sequence). *Let*$p=(p_{1},\ldots ,p_{k})$*be a path in the sequence graph*G=(V,E,σ)*; that is*, $p_{i}\in V$*for*i∈{1,…,k}*and*$(p_{i},p_{i+1})\in E$*for*i∈{1,…,k−1}*. Then, the path sequence of p, written*σ(p)*, is given by*$\sigma (p_{1})\sigma (p_{2})\cdots \sigma (p_{k})$.

 We note that this definition of paths and path sequences includes the possibility of repeated vertices: paths are allowed to visit the same vertex multiple times. In this article, we study two related graph problems: finding exact matches between a sequence and a path in a graph, termed sequence-to-graph matching (SGM) and the semi-global sequence-to-graph alignment (SGA) problem.

Problem 1 (Sequence-to-Graph Matching, SGM). *Let a string*$s\in \Sigma^{*}$and a sequence graphG=(V,E,σ)*be given. Find all paths*$p=(p_{1},\ldots ,p_{k})$*in G such that the path label*σ(p)*is equal to the string s, or report that such a path does not exist.*

Problem 2 (Unit Cost Semi-Global Sequence-to-Graph Alignment, SGA). *Let a string*$s\in \Sigma^{*}$and a sequence graphG=(V,E,σ)*be given. Find a path*$p=(p_{1},\ldots ,p_{k})$*in G such that the edit distance*d(σ(p),s)*is minimized and report a corresponding alignment of*σ(p)*and s*.

We assume a constant alphabet Σ. In the remainder of this article, we assume an arbitrary but fixed string $s\in \Sigma^{*}$ with |s|=m and sequence graph G=(V,E,σ) to be given. Without loss of generality, we assume that |V|≤2|E|+|Σ|. This can be assumed because, if |V|>2|E|, then there are nodes which are not connected to any other nodes. In this case, we can merge the disconnected nodes with the same label, producing a graph with at most 2|E|+|Σ| nodes.

## 3 Extending Shift-And to graphs

The *Shift-And* algorithm (Baeza-Yates and Gonnet, 1992; Baeza-Yates and Navarro, 1996; Dömölki, 1964, 1968) finds exact matches between a pattern string *s* of size *m* and a text string *t* of size *n*, with *m *<* n*, in $O\left(\left\lceil \frac{m}{w}\right\rceil n\right)$ time where *w* is the word size of the machine (usually 64 on modern computers). The Shift-And algorithm works by simulating a nondeterministic finite automaton (NFA) that matches the pattern, and then feeding the text to it. The state of the automaton is kept in a *m*-sized bitvector, consisting of $\left\lceil \frac{m}{w}\right\rceil$*w*-bit words, and the state is updated by shifting the vector by one and bitwise AND-ing the state with a precomputed character bitvector. The invariant of the algorithm is that the *i*’th bit in the NFA’s state is set after processing the *j*’th character in the text if and only if there is an exact match between the pattern prefix $s_{0..i}$ and the text substring $t_{j-i..j}$ (corresponding to a suffix of the text that has been processed so far). In this section, we generalize the Shift-And algorithm to graphs, starting with the simpler case of DAGs and then proceeding to general graph that may contain cycles. That is, we extend the Shift-And algorithm to solve SGM (Problem 1), which illustrates some of the concepts we later use in Section 4 to solve SGA (Problem 2).

### 3.1 Directed acyclic graphs

In DAGs, we process the nodes in topological order. If a node has an in-degree of 1, then the update proceeds in the same way as in the classical Shift-And algorithm: We use the previous automaton state (i.e. the state after processing the in-neighbor) and update it according to the label of the present node. However, some nodes have an in-degree of more than 1. For handling such nodes, we first propagate the NFA state from each in-neighbor separately. That is, we compute the updated state as if this node was the only in-neighbor. We then need to *merge* the resulting states such that any exact match from any in-neighbor translates to a match in the node. Here, the invariant to be maintained is that bit *i* in the bitvector representation of the NFA’s state is set after processing a given node if and only if there is a path of length *i* ending in this node and matching a length-*i* prefix of the pattern. Since the matching path can come from any of the in-neighbors, and a valid path from any of the in-neighbors translates to a valid path in the node, this invariant can be accommodated by merging the ‘incoming states’ using a bitwise OR operation. Since the merging is a $O\left(\left\lceil \frac{m}{w}\right\rceil \right)$-time operation, the overall time complexity is unchanged.

### 3.2 Cyclic graphs

The strategy for cyclic regions is similar to the previous one, except that, in the absence of a topological sorting, we process the nodes in an arbitrary order. The main idea to still arrive at correct values consists in storing a separate NFA state bit-vector for each graph node and to update them repeatedly until no more changes are necessary.

Algorithm 1 shows our algorithm as pseudocode. We keep a list of *calculable* nodes. All nodes are inserted into the calculable list at the start. Whenever a node is popped from the list, its state is propagated to its out-neighbors, and all out-neighbors whose state has changed are added to the list. A state change may set a bit but cannot unset a bit. Therefore, a node’s state may change up to *m* times, so each node may get added to, and popped from, the list up to *m* times. Each pop requires $O\left(\left|\delta_{x}^{\text{out}}\right|\right)$ time. The worst case runtime is therefore $O\left(\left|V|+m\Sigma_{x\in V}|\delta_{x}^{\text{out}}\right|\right)=O\left(\left|V|+m|E\right|\right)$. Correctness can be verified by observing that the above invariant must hold for all nodes once the calculable list is empty. Algorithm 1 can be simplified to the $O\left(\left|V|+\left\lceil \frac{m}{w}\right\rceil |E\right|\right)$ algorithm for DAGs by sorting *L* topologically, popping the nodes in order at Line 7, and removing the IF block starting from Line 11. For the DAG algorithm, we also do not need to keep the entire array *S*, but just a ‘frontier’ consisting of nodes whose out-neighbors have not been processed yet.

**Algorithm 1 Shift-And for cyclic graphs**

1: **Input**: a sequence graph (V,E,σ) and a string *s*

2: **Output**: Vector *S* containing the NFA states of *V*

3: P← precomputed pattern bitvectors for ∀c∈Σ based on *s*

4: L← a list initialized with V

5: S←|V|-sized array of integers initialized with 0

6: **while**|L|>0**do**

7:  v←L.pop()

8:  **for**$y\in \delta_{v}^{\text{out}}$**do**

9:   old←S[y]

10:  $S[y]\leftarrow S[y]\;\text{OR}\;(((S[v]\ll 1)+1)\;\text{AND}\;P_{\sigma (y)})$

11:  **if**S[y]≠old**then**

12:   L.push(y)

## 4 Extending Myers’ bitvector alignment to graphs

We approach SGA (Problem 2) by generalizing the standard DP algorithm for edit distance calculation. In our case, the DP matrix has one column per node $v_{i}\in V$ and one row per character *s_j_* from $s\in \Sigma^{*}$. We seek to compute values $C_{i,j}$ for i∈{1,…,|V|} and j∈{1,…,|s|} such that $C_{i,j}$ is the minimum edit distance d(p,s[1..j]) over all paths *p* ending in node *v_i_*.

Definition 3 (Recurrence for SGA). *Define*

(1) $$C_{i,j}=\min\;\{\begin{array}{cc} C_{k,j-1}+\Delta_{i,j}, & \text{for}\;k\in \delta_{i}^{\mathrm{in}}\\ C_{k,j}+1, & \text{for}\;k\in \delta_{i}^{\mathrm{in}}\\ C_{i,j-1}+1 & \end{array}$$

*with the boundary condition*$C_{i,1}=\Delta_{i,1}$*for all*i∈{1,…,|V|}*, where*$\Delta_{i,j}$*is the mismatch penalty between node character*$\sigma \left(v_{i}\right)$*and sequence character s_j_, which is 0 for a match and 1 for a mismatch*,

 We refer to the individual terms in Recurrence (1) as the *‘diagonal’* (topmost), *‘horizontal’* (middle) and *‘vertical’* (bottom) terms, due to their relative positions in the DP matrix. Despite cyclic dependencies in Recurrence (1), the problem has a unique solution for any graph and sequence; see the Supplementary Material for a proof. Recurrence (1) can be solved in O(|V|+m|E|) time (Navarro, 2000) in a *cell-by-cell* manner, where each operation calculates one individual cell. This is in contrast to Myers’ bitvector algorithm for sequence-to-sequence alignment which calculates multiple cells in a constant time operation (Myers, 1999).

 In linear sequence-to-sequence alignment, the recurrence implies the *vertical property* (Ukkonen, 1985), meaning that the score difference between two vertically neighboring cells is in the range {−1,0,1}, which is necessary for representing them using two *bitvectors* (Myers, 1999). To generalize Myers’ algorithm, we first establish that the vertical property also holds for graphs.

Theorem 1 (Vertical property for sequence-to-graph alignment). *The score difference between any two vertically adjacent cells*$C_{i,j}$*and*$C_{i,j-1}$*is at most one, that is*, $C_{i,j}-C_{i,j-1}\in \{-1,0,1\}$ for all i∈{1,…,|V|}*and*j∈{2,…,|s|}.

 The vertical property for graphs was implicitly proven by Navarro (2000) but not explicitly mentioned. The implicit proof assumes that the scores are first correctly calculated. However, in the bitvector algorithm the vertical property is a prerequisite to calculating the scores. Therefore, we give an alternate proof in the Supplementary Material which does not rely on this assumption.

### 4.1 Terminology

**Fig. 1.**
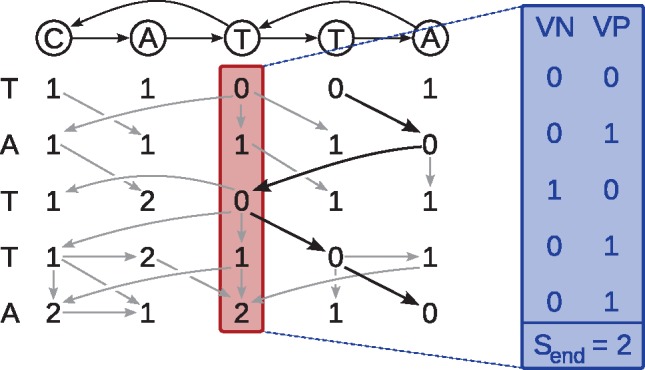
Dynamic programming matrix for aligning the sequence TATTA to the shown graph. Gray arrows indicate which predecessor cell(s) gave rise to the minimum value in Recurrence (1). Black bold arrows show the optimal path. As an example, the column highlighted in red is given in its bitvector representation (blue)

Figure 1 shows the relation between the concepts described here. The DP matrix is oriented with graph characters as columns and sequence characters as rows. A *column* in the DP matrix consists of *m* cells and corresponds to one node in the graph. We use the terms *column* and *node* interchangeably, depending on whether we are emphasizing the DP matrix or the graph topology. We use the term *calculating a column/node* to refer to the operation of using Recurrence (1) to process an edge and calculate the score of the edge’s destination column based on the edge’s source column and a character (the label of the destination node/column). The *minimum changed score* between two columns $C^{\text{old}}$ and $C^{\text{new}}$ is the minimum score of the *new* column at rows where the *new* column is smaller, that is, $\mathrm{minChanged}(C_{\text{old}},C_{\text{new}})=\mathrm{mi}n_{j\in [0,m):C_{\text{new},j}<C_{\text{old},j}}(C_{\text{new},j})$. If $C_{\text{new},j}\geq C_{\text{old},j}$ at every *j*, we say that the minimum changed score is infinite. The minimum changed score is used to distinguish cells which are relevant in cyclic areas; when recalculating a column, only those cells whose scores changed can propagate the scores onward.

We refer to the current DP table column we consider as *S*. Column *S* is stored in *bitvector representation* (Myers, 1999), consisting of a score $S_{\text{end}}$ attained in that column at the bottom row, a positive bitvector $VP^{S}$ and a negative bitvector $VN^{S}$, as illustrated in Figure 1 (blue box). The *word size w* is the number of bits in a computer word (usually 64). The positive and negative bitvectors consist of *m* bits and are implemented with $\left\lceil \frac{m}{w}\right\rceil$ machine words. For a column *S*, the *score* at index *j* is $S_{j}=\mathrm{popcount}(VP_{0..j}^{S})-\mathrm{popcount}(VN_{0..j}^{S})$, where *popcount* refers to the number of set bits in a bitvector. Note that $S_{\text{end}}=S_{m-1}$.

### 4.2 Directed acyclic graphs

**Fig. 2.**
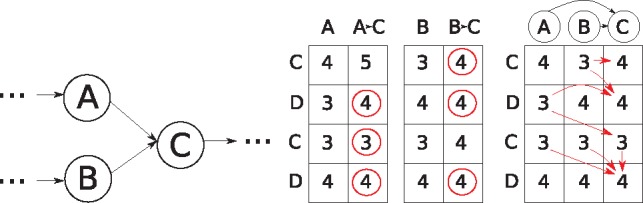
Handling nodes with an in-degree higher than one in the bitvector framework. Left: The node C has two in-neighbors, A and B. Middle: Each in-neighbor column is separately calculated to get the scores of Recurrence (1). The circled cells are the minimum of each row. Right: The resulting columns are merged, taking the minimum of the two scores for each row. The arrows show the possible backtraces for each cell

For DAGs, we use a similar strategy to the Shift-And algorithm. First we order the nodes topologically, and then we process the columns in order. However, Recurrence (1) now has terms for multiple in-neighbors. For handling nodes with an in-degree more than 1, we first calculate the incoming edge from each in-neighbor, that is, as if there was only one in-neighbor. Then, we *merge* the columns such that the cells of the resulting column have the minimum of each incoming column in that row: For two input columns *S^A^* and *S^B^*, we compute an output column *S^O^* such that $S_{i}^{O}=\min(S_{i}^{A},S_{i}^{B})$ for all indices *i*. Figure 2 shows an example of merging two columns. We defer the details of merging columns to Section 5, where we devise an algorithm to do this in $O\left(\left\lceil \frac{m}{w}\right\rceil \;\text{log}\;w\right)$ time. The operation must be applied at most *E* times. The runtime is therefore $O\left(V+\left\lceil \frac{m}{w}\right\rceil E\;\text{log}\;w\right)$.

### 4.3 Cyclic regions

Cell-by-cell algorithms for sequence-to-graph alignment (Myers and Miller, 1989; Navarro, 2000) handle cyclic dependencies in a row-wise manner: For each row, in a first sweep the ‘vertical’ and ‘diagonal’ terms of Recurrence (1) are calculated and, in a second sweep, the ‘horizontal’ terms are applied. However, this approach cannot be applied in a column-wise manner that is inherent to Myers’ bitvector algorithm. To deal with cyclic dependencies, we rely on two key ideas: First, we process the nodes in a specific order and, second, we recalculate scores of nodes until they have ‘converged’ (similar to our approach for the Shift-And algorithm).

**Algorithm 2 Bitvector alignment algorithm for cyclic graphs**

1: **Input**: a sequence graph (*V*, *E*) and a string *s*

2: **Output**: Vector *S* containing the column states of *V*

3: P← precomputed pattern bitvectors for ∀c∈Σ based on *s*

4: L← a priority queue initialized with (0,v),∀v∈V

5:S←|V|-sized array of bitvectors initialized with $\mathrm{VP}=1^{m},\mathrm{VN}=0^{m},S_{\text{end}}=m$

6: **while**|L|>0**do**

7:  (_,v)←L.pop()

8:  **for**$y\in \delta_{\mathrm{out}}^{v}$**do**

9:   old←S[y]

10:  ▹ ⊗: merge operation, *F*: bitvector step from Myers (1999)

11:  $S[y]\leftarrow S[y]⊗F(S[v],P_{\sigma (y)})$

12:  **if**changedMin(old,S[y])≠∞**then**

13:    L.push(changedMin(old,S[y]),y)

To define this order, we keep a priority queue of *calculable nodes* and their priorities. We define the operation *push*(*p*, *v*) for the priority queue: if the node *v* is not in the priority queue, *v* is inserted into the queue with the priority *p*; or if *v* is in the queue and *p* is smaller than *v’*s current priority, *v’*s priority is set to *p*; otherwise do nothing. Initially, all nodes are inserted into the queue with priority 0. All columns are initialized with a bitvector $\mathrm{VP}=1^{m},\mathrm{VN}=0^{m}$, corresponding to increasing scores. Then, nodes are picked from the queue in priority order (lowest first), and the out-neighbor columns are calculated based on the source column. For each out-neighbor *y*, the new column is merged with the existing column and the merged column is stored at *y*. Then, if the minimum changed value between the existing and the new column is not infinite, *y* is added to the calculable queue with the minimum changed value as the priority. Pseudocode is given in Algorithm 2. We use the symbol ⊗ to mark the column merging operation (see Section 5.2). We use the *F* to denote the column calculation operation from a predecessor column and a character match bitvector. This operation proceeds exactly like in Myers’ original bitvector algorithm and involves computing intermediate bitvectors for horizontal and diagonal differences. We do not discuss these details here and refer the reader to the original paper by Myers (1999) or to the textbook by Mäkinen *et al.* (2015). In the following, we will establish correctness and runtime of Algorithm 2.

We use the term *present scores* to refer to the scores assigned to the cells at some point during the calculation, as opposed to the *correct scores* which correspond to the unique scores that satisfy Recurrence (1). We say that a cell has *converged* when its present score is equal to its correct score.

Theorem 2. *In Algorithm 2, if the minimum priority of the calculable queue is x, then all cells whose correct scores are*$C_{i,j}<x$*have converged.*

Proof. We show this by induction. For the initial case, there are no cells whose correct scores are negative, so the statement holds when *x *=* *0. Next, we will assume that the minimum priority of the calculable queue is *x* and that all cells whose correct scores are $C_{i,j}<x-1$ have converged, and show that all cells whose correct scores are $C_{i,j}=x-1$ have converged. Assume that there is a cell whose correct score is *x* − 1. There are four cases for how the cell’s correct score is defined: (i) the vertical term, (ii) the horizontal term, (iii) the diagonal term with a mismatch, (iv) the diagonal term with a match.

*Case (i).* The cell has a vertical neighbor $C_{i,j-1}$ whose correct score is *x* − 2. By assumption cells with correct score $C_{i\prime ,j\prime }<x-1$ have converged, so the vertical neighbor’s present score is *x* − 2. The bitvector representation allows a vertical score difference of up to 1, so the cell’s present score is at most *x* − 1 and the cell has converged.

*Case (ii).* The cell has a horizontal neighbor $C_{i\prime ,j}$ whose correct score is *x* − 2. The neighbor cell has converged by assumption. After the last time the neighbor column was calculated, the neighbor cell had its correct score. Since there is a cell with a present score *x* − 2 in the neighboring column, the node i′ was added to the calculable queue with a priority of *x* − 2 (or less). Therefore, the edge (i′,i) was processed at some point earlier in the calculation, and at that point Recurrence (1) was applied to the cell $C_{i,j}$, producing the correct score.

*Case (iii).* Analogous to Case (ii).

*Case (iv).* The cell has a diagonal neighbor $C_{i\prime ,j\prime }$ whose correct score is *x* − 1. If the diagonal neighbor has converged, then the node i′ will have been added to the calculable queue with a priority of *x* − 1 (or less), and the argument from Case (ii) applies. Next we need to prove that the diagonal neighbor has converged. The diagonal neighbor cell’s correct score is again defined by the same cases (i)–(iv). For cases (i)–(iii), the diagonal neighbor has converged. For Case (iv), we look at the diagonal neighbor cell’s diagonal neighbor cell, and keep traversing by diagonal connections until we reach a cell for whom one of cases (i)–(iii) applies. Since the diagonal neighbors cannot form cycles, this will eventually happen, proving that the entire chain has converged.

 From Theorem 2, it follows that once the minimum priority of the calculable queue is *m *+* *1, all cells have converged to their correct scores, so the algorithm will eventually reach the correct solution in cyclic areas. Next we will establish an upper bound on the time until convergence.

Corollary 1. If all cells whose correct scores are $C_{i,j}<x$ have converged, then all cells whose present scores are $C_{i,j}\leq x$ have converged.

Proof. We assumed that all cells whose correct scores are $C_{i,j}<x$ have converged. Therefore, there are no cells whose present score is *x* but whose correct score is $C_{i,j}<x$. A cell’s present score cannot be lower than its correct score since the present scores are initialized at the highest possible value and applying Recurrence (1) cannot lower them under the correct score. Therefore, if a cell’s present score is *x*, it must also be its correct score.

Theorem 3. A node cannot be popped from the calculable queue more than *m* times.

Proof. If a node *v* is popped from the calculable queue with a priority *x*, it was added to the queue with a priority *x* at some point. This implies that there is at least one cell $C_{v,j}$ in the column with a present score of *x*. By Theorem 2 all cells with correct scores below *x* have converged and consequently $C_{v,j}$ has converged by Corollary 1. Therefore, each pop of a node *v* must be preceded by an update to node *v’*s state that causes at least one cell to converge. Since a cell can converge only once, and a column has *m* cells, this can happen at most *m* times per node.

 From Theorem 3, the outer loop starting in Line 6 runs at most m|V| times. Since the inner loop in Line 8 is processed $\left|\delta_{v}^{\mathrm{out}}\right|$ times per outer loop iteration, the inner loop runs at most $m\Sigma_{v\in V}|\delta_{v}^{\mathrm{out}}|=m|E|$ times. This provides a bound of m|E| inner loop iterations, meaning that in the worst case, the cyclic bitvector algorithm behaves like a cell-by-cell algorithm.

 Algorithm 2 uses a priority queue to store the calculable nodes. Since the maximum score a cell can have is *m*, the priority queue can be implemented as *m* arrays, one for each priority, plus a |V|-sized array for the node’s current position in the queue for the *push* operation. In this case, inserting and retrieving *n* values can be done in O(|V|+m+n) time. Since |V|≤n≤m|V|, this reduces to *O*(*n*) and the calculable queue has amortized constant time retrieval and insertion.

 In summary, the inner loop in Line 8 runs O(m|E|) times, while the runtime of each iteration depends on the implementation details, which we discuss below.

## 5 Bitvector implementation

The scores of each column are represented with a *bitvector* consisting of a positive bitvector VP, negative bitvector VN and score at end $S_{\text{end}}$. For a sequence of length *m*, the bitvectors consist of *m* bits, implemented as $\left\lceil \frac{m}{w}\right\rceil$ machine words. We use the term *elementary operation* to refer to arithmetic and bitwise operations (e.g. addition, subtraction, AND, OR) on all bits in parallel. For *m*-bit bitvectors, the elementary operations use $O\left(\left\lceil \frac{m}{w}\right\rceil \right)$ time. We use the term *column operation* to refer to higher level operations such as merging two bitvectors (⊗) and computing the minimum changed score (*changedMin*).

### 5.1 Slice-by-slice processing

**Fig. 3.**
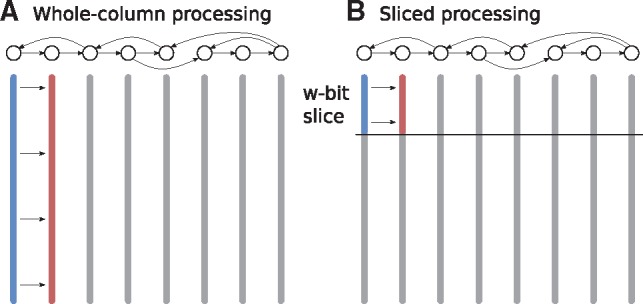
The DP table for aligning a sequence to a graph (shown on top) is represented by a set of columns (vertical bars), each corresponding to one graph node. The table can be filled in different orders: (**A**) each update operation (from blue to red) proceeds on a complete column. (**B**) Update operations commence on ‘slices’ of *w* bits; only after the final values in a slice (i.e. for all columns) have been computed, we proceed to the next slice

As outlined above, Algorithm 2 is designed to update one column at a time through bit-parallel column operations. Alternatively, it is possible to ‘slice’ the DP table into pieces of *w* rows each, as illustrated in Figure 3. If we slice the bitvector into *w*-bit slices, elementary operations can be performed in *O*(1) time within a slice. The idea is to apply Algorithm 2 separately to each of the $\left\lceil \frac{m}{w}\right\rceil$ slices, proceeding from top to bottom. To accommodate this, we need to carry over the bottom most values in a slice into the next slice. To this end, we add an extra variable *score before start*$S_{\text{before}}$ to each bitvector, which is 0 for the topmost slice and equal to the above slice’s $S\prime_{\text{end}}$ for other slices. While it does not change the results, this sliced processing will allow us to speed up the total runtime.

### 5.2 Bitvector merging algorithm

When merging two columns (operation ⊗), we are given two input columns *S_A_* and *S_B_*, represented in memory through ($VP^{A},VN^{A},S_{\text{before}}^{A},S_{\text{end}}^{A}$) and ($VP^{B},VN^{B},S_{\text{before}}^{B},S_{\text{end}}^{B}$). As output, we seek to compute ($VP^{O},VN^{O},S_{\text{before}}^{O},S_{\text{end}}^{O}$), the bitvector representation of a column *S_O_* such that its values are the minimum of the two columns represented by the input bitvectors, that is, $S_{i}^{O}=\min(S_{i}^{A},S_{i}^{B})$ for all i∈{0,1,…,m−1}.

**Fig. 4.**
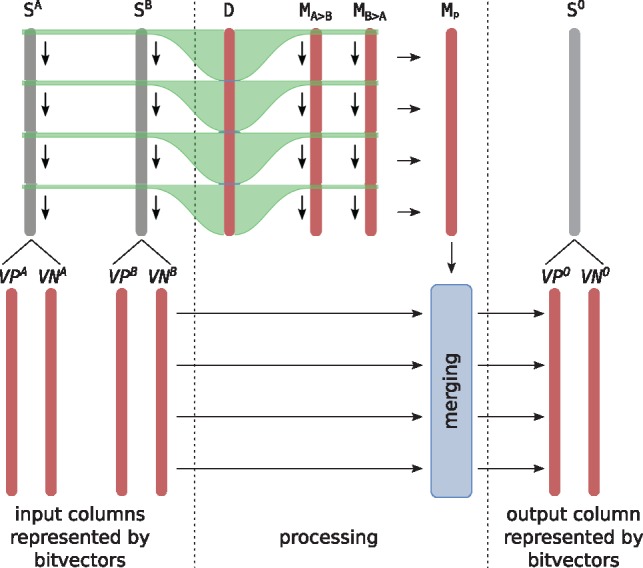
Conceptual idea of bit-vector merging. Red bars represent bit-vectors, which are stored in memory. Gray bars represent input/output columns, which are never stored explicitly, but represented implicitly by the respective bit-vectors. The variable *D* is split into chunks of size O(log m), where the bits in each chunk encode the difference between a particular row in *S^A^* and *S^B^*, as indicated by green lines. The values in each chunk are used to compute the respective bits in $M_{A>B}$ and $M_{B>A}$. In each iteration, the chunks are updated to represent a difference of *S^A^* and *S^B^* one row further down, indicated by down arrows. Once $M_{A>B}$ and $M_{B>A}$ have been computed, the ‘picking mask’ *M_p_* is computed in parallel (horizonal arrows) and used in the final merging step (blue box)

The overall concept of our merging algorithm is illustrated in Figure 4, while we present pseudo code, a detailed example and an extended discussion of implementation details in the Supplementary Material. The key idea consists in computing the difference between entries in *S_A_* and *S_B_* in parallel as follows: We define a variable *D* split in *chunks* of ${\;\text{log}\;}_{2}m+2$ bits, where each chunk represents the score difference $S^{A}-S^{B}$ at a certain index, as illustrated by the green lines in Figure 4. Updating *D* such that each chunk now represents a difference value for the next row can then be done in constant time, processing all chunks in parallel. In this way, we consecutively compute entries in two *difference bit masks*$M_{A>B}$ and $M_{B>A}$, which indicate rows where the score of *A* is higher than *B* and vice versa, respectively. Once $M_{A>B}$ and $M_{B>A}$ have been computed, we can, again in parallel, compute a *picking mask M_p_*, which essentially tells us which values have to be picked from $\left(VP^{A},VN^{A}\right)$ and which have to be picked from $\left(VP^{B},VN^{B}\right)$ to compute the final output bitvectors $\left(VP^{O},VN^{O}\right)$.

We need O(log m) iterations to compute $M_{A>B}$ and $M_{B>A}$, each of which uses a constant number of elementary operations. Computing *M_p_* as well as the final merging also take a constant number of elementary operations, each of which takes $O\left(\left\lceil \frac{m}{w}\right\rceil \right)$ time (see above). Therefore, we need a total of $O\left(\left\lceil \frac{m}{w}\right\rceil \;\text{log}\;m\right)$ time to merge two bitvectors.

### 5.3 Changed minimum value algorithm

The *changed minimum value* of two bitvectors *old* and *new* is the minimum value at indices where the new bitvector has a smaller value than the old, that is, $\mathrm{changedMin}(\mathrm{old},\mathrm{new})=\mathrm{mi}n_{i:S_{i}^{\mathrm{new}}<S_{i}^{\mathrm{old}}}(S_{i}^{\mathrm{new}})$. The changed minimum value can be calculated in O(log w) time by splitting the bitvector into chunks and calculating the value at each log w’th position in parallel, similarly to the difference mask algorithm. However, in practice it is faster to calculate the difference mask $M_{\mathrm{new}<\mathrm{old}}$ and find all local minima where $S_{i}^{\mathrm{new}}<S_{i}^{\mathrm{old}}$ by using the VP, VN and $M_{\mathrm{new}<\mathrm{old}}$ vectors. An index is a local minimum if VP is set to its left (more significant bits) and VN is set either to its right (less significant bits) or at the index. Then, each local minimum is processed one at a time. The score at the index is calculated using the definition of the implied scores $S_{i}=\mathrm{popcount}(VP_{0..i})-\mathrm{popcount}(VN_{0..i})$. This takes *O*(*w*) time but in practice there are very few local minima, leading to a speedup over the O(log w) algorithm.

### 5.4 Asymptotic runtime

Algorithm 2 executes its inner loop (Line 8) to update a column O(m|E|) times (see Section 4.3). The two *column operations* of merging two bitvectors and computing the minimum changed score use O(log k)*elementary operations* for a bitvector of *k* bits. When processing a whole column (i.e. *k *=* m*), then this leads to a runtime of $O\left(\left\lceil \frac{m}{w}\right\rceil \;\text{log}\;m\right)$ for each column operation and to $O\left(|V|+m|E|\left\lceil \frac{m}{w}\right\rceil \;\text{log}\;m\right)$ in total. When processing the DP table in slices (Fig. 3), we need to run Algorithm 2 once for each slice, that is, $\left\lceil \frac{m}{w}\right\rceil$ times. Processing each slice will lead to O(w|E|) update operations in Line 8, each of which takes O(log w) time. In total, we can hence compute the full DP matrix in O(|V|+m|E| log w) time. Like the Shift-And algorithm, the cyclic algorithm can also be simplified for DAGs by ordering *L* topologically in Line 4 and removing the IF-block starting at Line 12, producing an $O\left(|V|+\left\lceil \frac{m}{w}\right\rceil |E|\;\text{log}\;w\right)$ algorithm.

## 6 Experiments

We implemented the sequence-to-graph bitvector algorithm described here and the cell-by-cell algorithm by Navarro (2000). We performed several experiments on the algorithms: the *bitvector performance experiment*, comparing our approach to existing well-optimized implementations of Myers’ algorithm on a linear sequence; the *graph topology experiment*, comparing the effect of different graph topologies; the *HLA experiment*, measuring the speedup on a more realistic use case; and finally, the *Escherichia coli experiment* aligning reads to a graph resulting from genome assembly. The source code of the experiments is available at https://github.com/maickrau/GraphAligner/tree/PaperExperiments.

### 6.1 Bitvector performance

The sliced processing (Fig. 3) adds extra overhead compared with the whole-column processing used in the classical Myers’ algorithm. The reference sequence must be accessed multiple times, and memory use is not cache-efficient, since a large memory range is written and read a few times per address instead of a small range updated many times per address. To measure the overhead added by this, we ran the bitvector algorithm on a graph consisting of a linear chain of nodes with 200 000 bp in total and a 100 000 bp query. This linear graph mimicks sequence-to-sequence alignment and we compared our performance with optimized implementations of Myers’ algorithm from BGSA (Zhang *et al.*, 2018) and Seqan (Döring *et al.*, 2008) on the same sequences. We also tested whole-column processing for the linear graph to see how much of the difference is due to code optimization and how much is due to the different processing methods. Note that BGSA is particularly designed to be fast in the case when multiple reads are aligned in parallel. To facilitate a meaningful comparison, we used BGSA in a mode resembling Myers’ bitvector algorithm, that is, we aligned one read on one CPU without using vector instructions.

Table 1 shows the results. The sliced processing method is noticably slower than the optimized implementations or the whole-column method. The whole-column method’s performance is close to the optimized implementations, which indicates that our implementation does not incur significant overheads. Unfortunately, the whole-column method is slow in graphs with nodes with in-degree two or more due to the merge operation’s performance. The overhead of the sliced processing method therefore seems to be inherent to processing non-trivial graphs. In the remaining experiments we use the sliced processing method.

**Table 1.** Sliced versus whole-column processing on a linear graph

BGSA	Seqan	Our method (whole-column)	Our method (sliced)
1.3s	1.2s	1.5s	5.5s

### 6.2 Graph topology experiment

**Fig. 5.**
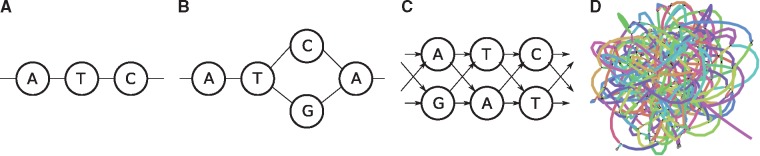
Overview of the graphs used in the graph topology experiment. (**A**) Linear graph, (**B**) SNP graph, (**C**) twopath graph, (**D**) tangle graph [visualized with Bandage (Wick *et al.*, 2015)]

For the graph topology experiment, we created four kinds of graphs (Fig. 5), representing increasing levels of difficulty, based on the *E.coli* reference genome’s 10 000 first base pairs.

The first graph, the *linear graph*, is a linear chain of nodes. Aligning to this graph is equivalent to sequence-to-sequence alignment. The second graph, the *SNP graph*, is a linear chain of nodes with randomly inserted bubbles representing single nucleotide polymorphisms (SNPs). The SNPs are distributed at an average of one SNP per 10 base pairs. The third graph, the *twopath graph*, is an artificial worst case graph for the bitvector algorithm. Each node has two in-neighbors, which means that the O(log w) bitvector merging algorithm has to run for each node. For the first three graphs, neither algorithm’s runtime depends on the matched sequence, so the additionally inserted nodes were given random labels. The fourth graph, the *tangle graph*, is based on a de Bruijn graph of the reference sequence with *k *=* *11. We chose *k* to be so small specifically to make the graph very cyclic and tangled.

For the tangle graph, the non-branching areas are merged to unitigs, and overlaps between the nodes are removed by deleting the last *k* − 1 characters of each non-tip node, producing a directed node-labeled graph with the same topology and same paths as the original de Bruijn graph. For each graph, we also included the reverse-complement strand to map reads simulated from the backwards strand, doubling the graph size and effectively mimicking a bidirectional graph. The graph sizes in Figure 5 refer to this doubled bidirectional size.

We simulated reads with 20× coverage (total 200 000 bp) from the reference using PBSIM (Ono *et al.*, 2013), which produced 65 reads with an average length of 3 kbp. In addition, we took a high coverage Illumina dataset (https://www.ebi.ac.uk/ena/data/view/ERX008638), filtered the reads by using minimap2 (Li, 2018) to select reads which align to the first 10 000 bp of the reference, and then randomly sampled a 50.5× coverage subset (5050 reads, 505 000 bp). Then, we aligned both the simulated long reads and the real short reads to the graphs using both our bitvector algorithm and the cell-by-cell approach.

### 6.3 HLA-A experiment

To assess the algorithm’s performance on a more realistic scenario, we built a graph of the human HLA-A gene and aligned real sequencing data to it. We took the 4637 alleles of the human HLA-A gene available from the IMGT/HLA database (Robinson *et al.*, 2015), and computed a multiple sequence alignment between them by using Clustal Omega (Sievers et al., 2011) version 1.2.4 with the command ‘clustalo -i sequences.fasta –outfmt clustal > aln.clustal’. Then we used vg (Garrison *et al.*, 2018) version 1.9.0 to build a variation graph from the multiple sequence alignment with the command ‘vg construct -M aln.clustal -F clustal -m 32 > msa.vg’.

For the sequence data, we used Illumina and PacBio reads from NA19240 (Chaisson *et al.*, 2018). To filter the Illumina reads, we used minimap2 (Li, 2018) to align the reads to the known alleles, producing 2829 Illumina reads (355 981 bp) with an alignment, which we considered to be from the HLA-A region. For the PacBio reads, we selected those whose alignment to the reference genome overlaps with HLA-A’s location, producing 102 reads (405 415 bp). Both the Illumina and PacBio reads were then aligned to the graph using the bitvector and cell-by-cell algorithms.

### 6.4 *Escherichia coli* experiment

For the *E.coli* experiment, we used sequencing data of *E.coli* strain K-12 substrain MG1655. We took 670× coverage Illumina reads from the European Nucleotide Archive (https://www.ebi.ac.uk/ena/data/view/ERX008638) and 144× coverage PacBio reads from the NCBI sequence archive (https://trace.ncbi.nlm.nih.gov/Traces/sra/?run=SRR1284073). We built a de Bruijn graph of the Illumina dataset using BCalm (Chikhi *et al.*, 2016), with *k* = 31 and *k*-mer solidity threshold 7. We applied the same postprocessing of the graph as described above for the tangle graph. Then we selected PacBio reads longer than 1000 base pairs and randomly sampled a subset of them corresponding to 1.5× average genome coverage, and aligned them to the graph with the bitvector and cell-by-cell algorithms.

### 6.5 Results

Table 2 shows a summary of the results. The first eight rows correspond to the graph topology experiment and the last three to the HLA-A and *E.coli* experiments. Each number is an average over 10 runs, showing the total time to align all reads on one CPU core of an Intel Xeon E7-8857 v2 CPU running at 3GHz. The bitvector approach is faster than the cell-by-cell approach in each graph. As expected from the time complexity analysis, the difference is greater in the acyclic graphs. For the acyclic graphs, the bitvector algorithm achieves between 10-fold and 20-fold speed improvement. For the cyclic graph, the speedup is between 3-fold and 5-fold, suggesting that cycles are recalculated on average only a few times (linear speedup divided by cyclic speedup) instead of the theoretical worst case of *w* times. The HLA-A and *E.coli* experiments show that the results generalize to more realistic scenarios as well. Note that in our experiments, we compute the *complete* DP matrix, and therefore, the long absolute time for the *E.coli* experiment are not surprising. In fact, this shows the feasibility of computing *optimal* alignments for bacterial genomes.

**Table 2.** Experimental results

Graph	Reads	Nodes	Edges	Bitvector	Cellwise	Speedup
Linear	PBSIM	20 000	19 998	1.2s	23.5s	19.6×
Linear	Illumina	20 000	19 998	5.5s	62.5s	11.4×
SNP	PBSIM	22 030	24 058	2.3s	41.8s	18.5×
SNP	Illumina	22 030	24 058	9.0s	106s	11.8×
Twopath	PBSIM	40 004	80 000	13.0s	168s	12.9×
Twopath	Illumina	40 004	80 000	42.1s	446s	10.6×
Tangle	PBSIM	19 814	20 398	8.1s	39.4s	4.8×
Tangle	Illumina	19 814	20 398	33.8s	102s	3.0×
HLA-A	PacBio	5864	9668	2.4s	51.0s	21.3×
HLA-A	Illumina	5864	9668	3.7s	44.5s	12.1×
*Escherichia coli*	PacBio	10 510 252	10 540 270	156 000s	1 860 000s	11.9×

## 7 Discussion

In this article, we generalized two sequence-to-sequence algorithms to sequence-to-graph algorithms. For the Shift-And algorithm, the runtime for acyclic graphs matches the runtime of the linear version, and the runtime for cyclic graphs matches cell-by-cell comparison algorithms for graphs. For the bitvector alignment algorithm, the runtime includes an extra log w term due to the complexity of merging bitvectors and finding the changed minimum value. Despite the graph-based bitvector alignment algorithm’s higher worst case time complexity compared with previous cell-by-cell alignment algorithms, it still achieves a 3-fold to 20-fold speedup over cell-by-cell algorithms depending on the shape of the graph. Should an algorithm for merging bitvectors and finding the changed minimum score in *O*(1) time exist, that would lead to the bitvector graph algorithm being asymptotically faster than cell-by-cell algorithms as well.

Our algorithm is defined with unit costs for mismatches and indels. Other approaches have extended bit-parallelism to generalized integer costs (Loving *et al.*, 2014; Zhang *et al.*, 2018). Using generalized integer costs with our graph-based approach would require extending the column merge and changed minimum value operations to the different score representation used by the generalized integer cost algorithms. The time complexity of the algorithm might also change due to the priority queue if the scores are not bounded by a reasonably small number.

Affine gap penalties (Gotoh, 1982) are commonly used in linear sequence alignment. This is implemented by adding two extra matrices, one for insertions and another for deletions. The same method can be used for cell-by-cell graph alignment by including two extra copies of the graph (Rautiainen and Marschall, 2017). We believe that this can also be applied to the bit-parallel version of graph alignment. This would require extending the scoring method to generalized integer costs, as otherwise the gap open and gap extend parameters would be one, defeating the whole point of using affine gap penalties. The extra subgraphs would also require more convergence analysis to determine the effect on runtime.

The bitvector algorithm described here provides a basis for practical algorithms for fast sequence-to-graph alignment. We believe that it can be scaled to mammalian genome sizes when combined with strategies for banded alignment.

## Supplementary Material

btz162_Supplementary_Material
