# Supplementary material for: Bit-parallel sequence-to-graph alignment
Source: Bioinformatics. 2019 Mar 9;35(19):3599–607. doi: 10.1093/bioinformatics/btz162 (PMC6761980; doi:10.1093/bioinformatics/btz162)
Supplement: btz162_Supplementary_Material [file btz162_supplementary_material.pdf]

Supplementary Material: Bit-parallel sequence-to-graph alignment

Mikko Rautiainen, Veli Mäkinen, and Tobias Marschall

A. Existence and uniqueness of a solution for Recurrence 1

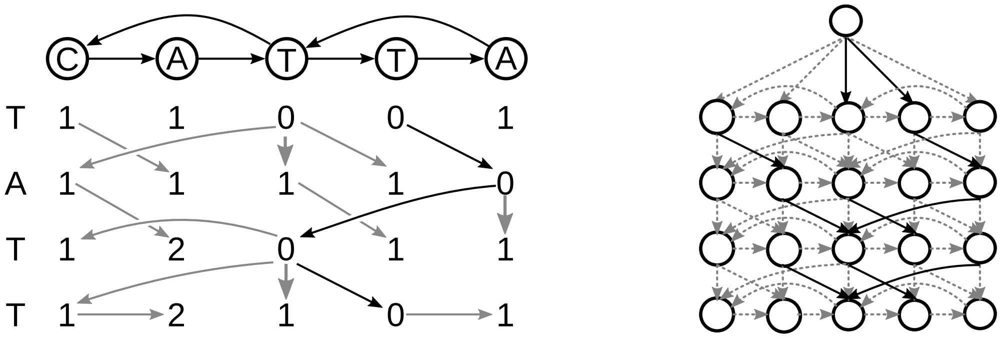

Figure 1: Left: the DP matrix of the alignment between a sequence graph (top) and a sequence (left). The gray arrows are the backtrace and the solid black arrows show the optimal alignment. Right: the DP graph for the same sequence graph and sequence. The dashed grey edges have a cost of 1, and the solid black edges have a cost of 0. The source node v_A^{source} is at the top. The distance from the source node to any node is equal to the score in the DP matrix.

Recurrence 1 cannot be directly used to calculate the scores for cyclic regions. Instead, we consider this recurrence a constraint on the cell scores, and then find an assignment of scores to the cells which satisfies the recurrence. There exists exactly one assignment of scores that satisfies the recurrence, which we prove in the following. To this end, we employ a classic relationship between edit distances and shortest path

problems: we define an *alignment graph* with one node for each cell in our DP table, such that the distance from a virtual source node equals the value in the corresponding cell (Myers, 1991), as illustrated in Figure 1(right). Formally, the alignment graph (not to be confused with a sequence graph) is defined as follows.

Definition 1 (Alignment graph). *Given a string $s \in \Sigma^m$ and sequence graph $G = (V, E, \sigma)$ with $V = \{v_1, \ldots, v_n\}$, we define the corresponding* alignment graph G_A *through a node set $V_A := V \times \{1, \ldots, |s|\}$ and a weight function*

$$w_A : \big((v_k, j), (v_i, j')\big) \mapsto \begin{cases} \Delta_{i,j} & (v_k, v_i) \in E \text{ and } j+1 = j', \\ 1 & (v_k, v_i) \in E \text{ and } j = j', \\ 1 & v_i = v_k \text{ and } j+1 = j', \\ \infty & \text{otherwise,} \end{cases}$$

where an edge exists between two nodes from V_A if the corresponding weight is finite. Additionally, we add a special node v_A^{source} and $\Delta_{i,1}$-weight edges from v_A^{source} to every node $(v_i, 1)$.

Theorem 1 (Existence). *For any sequence graph $G = (V, E, \sigma)$ and sequence $s \in \Sigma^m$, there exists an assignment of cell scores $C_{i,j}$ that satisfies the recurrence given in Recurrence 3.*

Proof. We consider the corresponding alignment graph. We observe that none of its edges can go "up", that is, for any edge $(v_k, j) \to (v_i, j')$, we always have $j \leq j'$ by Definition 1. Hence, all cycles will be among nodes in the same "row" (i.e. for the same value of j). Such "horizontal" edges with $j = j'$ have a weight of 1 by definition of the weights. Together, this implies that all cycles have a positive, non-zero cost. That, in turn, implies that the minimum distance from the source node v_A^{source} to any other node is well-defined and unique. We set $C_{i,j}$ to the minimum distance from v_A^{source} to (v_i, j). This assignment of $C_{i,j}$ values satisfies the recurrence in Definition 3, which follows immediately by induction since weights in Definition 1 mirror Recurrence (1). □

Theorem 2 (Uniqueness). *For any sequence graph $G = (V, E, \sigma)$ and sequence $s \in \Sigma^m$, there is at most one assignment of cell scores $C_{i,j}$ that satisfies Definition 3.*

Proof. The existence of a solution was established in Theorem 1 and we denote the cell scores given by the shortest paths in the corresponding alignment graph by $C_{i,j}$. Suppose, for the sake of contradiction, that there exists a different assignment $C'_{i,j}$ for $i \in \{i, \ldots, |V|\}$ and $j \in \{1, \ldots, |s|\}$. Let i' and j' be indices such that $C_{i',j'} \neq C'_{i',j'}$. Now consider a shortest path from v_A^{source} to (i', j'), corresponding to a sequence of nodes $\big(v_A^{\text{source}}, (i_1, j_1), \ldots, (i_n, j_n)\big)$ with $(i_n, j_n) = (i', j')$. Let k be the smallest index such that $C_{i_k,j_k} \neq C'_{i_k,j_k}$. Note that $k > 1$ because the initialization of the first row of the DP table (Definition 3) is identical to the choice of weights of edges from the source node v_A^{source} to the first row of vertices (Definition 1), and hence $C_{i_1,j_1} = C'_{i_1,j_1}$. If $C_{i_k,j_k} < C'_{i_k,j_k}$, then C'_{i_k,j_k} violates Recurrence (1), because Recurrence (1) minimizes over all possible predecessor cells, which correspond exactly to the incident

nodes of (i_k, j_k) in the alignment graph and the term in Recurrence (1) containing $C'_{i_{k-1},j_{k-1}}$ hence gives rise to a smaller value—a contradiction. If $C_{i_k,j_k} > C'_{i_k,j_k}$, then $\left(v_A^{\text{source}}, (i_1, j_1), \ldots, (i_k, j_k)\right)$ cannot be a shortest path from v_A^{source} to (i_k, j_k), because a shorter path can be obtained by following the "backtrace" through the DP table, i.e. by following the sequence of cells given by the minima picked in Recurrence (1). □

B. Proof of Theorem 1 (Vertical property)

Proof. It is clear from Recurrence 1 that $C_{i,j} - C_{i,j-1} \leq 1$. Next, we have to prove the bound $C_{i,j} - C_{i,j-1} \geq -1$ or, equivalently, $C_{i,j-1} \leq C_{i,j} + 1$. We distinguish three cases, based on which of the three terms terms in Recurrence 1 takes a minimum (note that more than one term can have the minimum value).

Case 1 (vertical, $C_{i,j} = C_{i,j-1} + 1$). This case immediately implies $C_{i,j} - C_{i,j-1} \geq -1$.

Case 2 (diagonal, $C_{i,j} = C_{k,j-1} + \Delta_{i,j}$ for $k \in \delta_i^{in}$). By Recurrence (1), we have $C_{i,j-1} \leq C_{k,j-1} + 1$. Therefore, $C_{i,j-1} \leq C_{k,j-1} + 1 = C_{i,j} - \Delta_{i,j} + 1$ by the assumption of Case 2. It follows $C_{i,j} \geq C_{i,j-1} + \Delta_{i,j} - 1$, which implies $C_{i,j} - C_{i,j-1} \geq -1$ since $\Delta_{i,j} \geq 0$ by definition.

Case 3 (horizontal, $C_{i,j} = C_{k,j} + 1$ for $k \in \delta_i^{in}$). Our proof for this case is by induction on values of the cells in a row. That is, to prove the claim $C_{i,j-1} \leq C_{i,j} + 1$ for cell $C_{i,j}$, we assume that it holds for all cells in the same row with smaller value, i.e. for all cells $C_{i',j}$ with $C_{i',j} < C_{i,j}$. Note that the claim holds for all cells with minimum value in each row, because they are covered by Case 1 or Case 2, but cannot fall under Case 3. By the induction hypothesis, we have $C_{k,j-1} \leq C_{k,j} + 1$ and, by the assumption of Case 3, we get $C_{k,j-1} \leq C_{i,j}$. By Recurrence 1, we have $C_{i,j-1} \leq C_{k,j-1} + 1$. Together, this implies that $C_{i,j-1} \leq C_{k,j-1} + 1 \leq C_{i,j} + 1$. □

C. Details of bitvector merging algorithm

Figure 2 contains a running example of merging two bitvectors with an $O(\log w)$ algorithm, which we use to explain the algorithm. For completeness, we provide full pseudocode below as Algorithm 1 on Page 7 and refer to the respective line numbers below.

The input for the bitvector merging algorithm are two bitvectors A and B, consisting of values VP^A, VN^A, S^A_{before}, S^A_{end} and VP^B, VN^B, S^B_{before}, S^B_{end} (step A). We assume that $S^A_{before} \leq S^B_{before}$. These bitvectors implicitly represent the values S^A_i and S^B_i (step B). The output is the bitvector representation $(VP^O, VN^O, S^O_{before}, S^O_{end})$ of a column S_O such that its values are the minimum of the two columns represented by the input bitvectors, that is, $\forall i \in \{0, 1, ..., w-1\} : S^O_i = \min(S^A_i, S^B_i)$ (step C).

First, we need to find *difference masks* $M_{A>B}$ and $M_{B>A}$, which describe cells where the score of A is higher than B and vice versa (step D). To do this, we first verify that the score differences $S^A - S^B$ are in the range $(-2w, 2w)$ (lines 24-29). We need the *popcount* operation for this; in most processors an $O(1)$ specialized instruction exists,

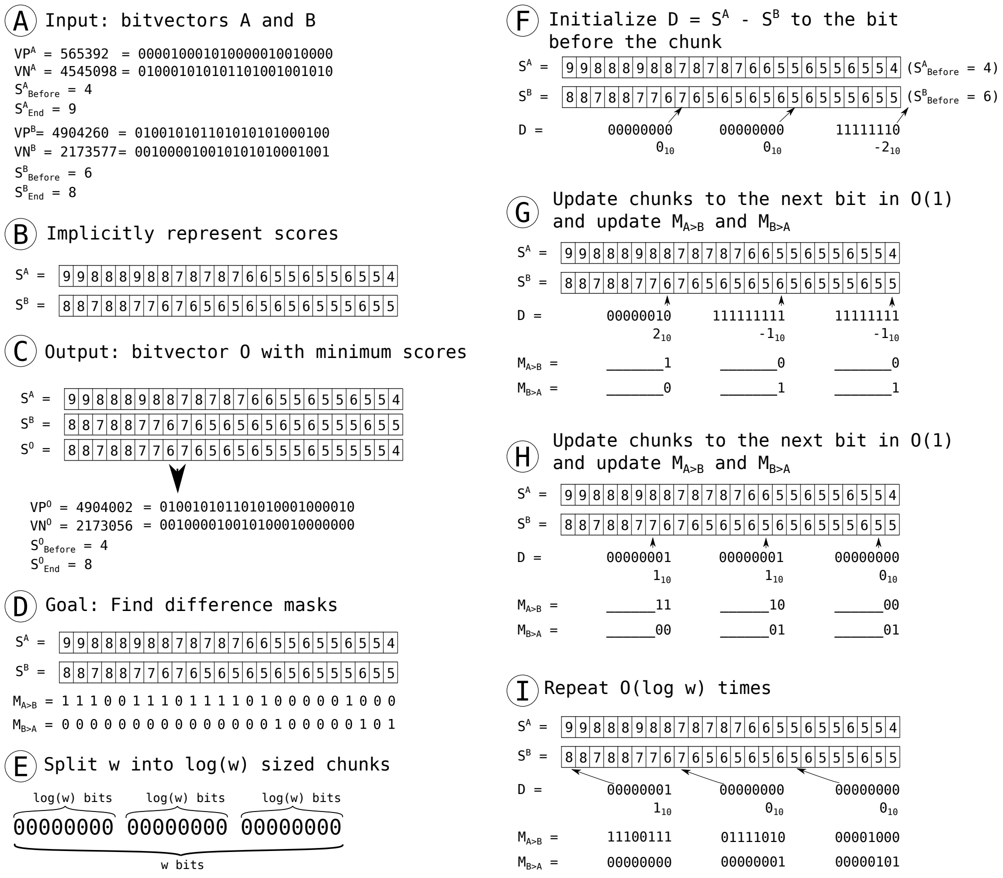

Figure 2: Overview of the bitvector merging algorithm

otherwise it can be calculated in $O(\log w)$ with normal arithmetic operations. We define a variable D split in *chunks*, where each chunk represents the score difference $S^A - S^B$ at a certain index. Each chunk is represented by $\log_2 w + 2$ bits and stores a value in two's complement. In this way, each chunk can accommodate a score difference, which takes a value between $-2w$ and $2w$. The chunks essentially simulate a SIMD-like architecture, with the chunks corresponding to SIMD registers, and parallel addition, substraction and comparison to zero instructions implemented with normal arithmetic and bitwise operations. This enables calculating the score difference $S^A - S^B$ in parallel. The chunks of D are first initialized to point at the bit before the start of the chunk (step F, lines 30-34).

Then D is updated in parallel to point at the next bit (step G, lines 36-37). The bitvectors VP^A, VP^B, VN^A and VN^B represent the score difference between two cells, and are used to update D. Since D represents the current score difference $S^A - S^B$, we can update it by $D' = S^A_{i+1} - S^B_{i+1} = S^A_i - S^B_i + (S^A_{i+1} - S^A_i) - (S^B_{i+1} - S^B_i) = D + (VP^A_{i+1} - VN^A_{i+1}) - (VP^B_{i+1} - VN^B_{i+1})$. This is implemented by shifting the

appropriate bits from the VP and VN bitvectors to the start of the chunk and selecting the least significant bit, essentially initializing the chunk-registers as either 0 or 1, which are then added and substracted to D (step G, lines 36-37).

Once D has been updated to the next bit, $M_{A>B}$ and $M_{B>A}$ can be updated based on the values in the chunks of D (step G, lines 38-41). Since the chunks of D represent the score difference, $M_{A>B}$ must be set at indices where the value of D is greater than 0, and $M_{B>A}$ where the value is less than 0. As the values are stored in two's complement, the case $D < 0$ is checked by selecting the sign bit and shifting it to the current position (lines 38 and 40). To check $D > 0$, we select indices where D is not negative and not zero (lines 39 and 41).

Updating D, $M_{A>B}$ and $M_{B>A}$ is then repeated $O(\log w)$ times to solve the full masks $M_{A>B}$ and $M_{B>A}$ (step I, lines 35-41). Initializing the variable D takes $O(\log\log w)$ time due to the chunk popcounts in line 6. Each iteration of the parallel update takes $O(1)$ time, and there are $O(\log w)$ iterations. The runtime of calculating the difference masks is therefore $O(\log w)$.

Once we have the difference masks $M_{A>B}$ and $M_{B>A}$, we can use them to merge the bitvectors. Given an index i, if $S_i^A \geq S_i^B$ and $S_{i-1}^A \geq S_{i-1}^B$, then the output bitvectors can be selected as $VP_i^O = VP_i^A$ and $VN_i^O = VN_i^A$, and vice versa if $S_i^B \geq S_i^A$ and $S_{i-1}^B \geq S_{i-1}^A$. However we need to handle the case where $S_i^A > S_i^B$ and $S_{i-1}^B > S_{i-1}^A$ or symmetrically $S_i^B > S_i^A$ and $S_{i-1}^A > S_{i-1}^B$. In this case, $S_i^A = S_{i-1}^B$, $VN_i^A = 1$, $VP_i^B = 1$ and $VP_i^O = VN_i^O = 0$. We first calculate a picking mask M_p which determines whether a bit needs to be taken from S^A (line 46). The picking mask is set to 1 whenever $M_{A>B} = 1$ and 0 to whenever $M_{B>A} = 1$, and in other indices copies the neighboring bit from the least significant direction. Then we pick the values for VP^O and VN^O based on the picking mask. To handle the special case where $S_i^A > S_i^B$ and $S_{i-1}^B > S_{i-1}^A$, we reduce the VN^A and VN^B vectors such that in those indices the output bitvector's value cannot decrease (lines 47-48). Merging bitvectors, given the difference masks, is $O(1)$. The total runtime is therefore $O(\log w)$.

However, in practice it is faster to precompute the difference masks for each possible 8-bit combination of $S_{before}^B - S_{before}^A$, VP^A, VP^B, VN^A and VN^B. Now, finding the difference masks takes $\frac{w}{8}$ memory lookups. With the lookup, merging is $O(w)$ but faster in practice than the $O(\log w)$ algorithm.

References

Myers, E. W. (1991). An overview of sequence comparison algorithms in molecular biology. Technical Report 91-29, Department of Computer Science, University of Arizona.

Algorithm 1 The bitvector merging algorithm

$chunkSize \leftarrow (\log_2 w) + 2$ rounded up to the nearest power of two
$M_{lsb} \leftarrow$ a constant mask which has 1 at each chunk's least significant bit and 0 elsewhere
$M_{sign} \leftarrow$ a constant mask which has 1 at each chunk's sign bit and 0 elsewhere

function CPFS$(x, extra)$ ▷ Chunk prefix sums
  $x \leftarrow$ popcount for each $chunkSize$-bit block
  $x \leftarrow (x \ll chunkSize) + extra$
  $x \leftarrow x * M_{lsb}$ ▷ x now has the prefix sum of the set bits at each chunk boundary
  return x

function AddC(x, y) ▷ Calculates the values $x + y$ at each chunk in parallel
  $signs \leftarrow x \ \& \ M_{sign}$
  $x \leftarrow ((x \ \& \sim M_{sign}) + y) \wedge signs$
  return x

function DeductC(x, y) ▷ Calculates the values $x - y$ at each chunk in parallel
  $signs \leftarrow x \ \& \ M_{sign}$
  $x \leftarrow (x|M_{sign}) - y$
  $signs \leftarrow signs \wedge (M_{sign} \ \& \sim x)$
  $x \leftarrow (x \ \& \sim M_{sign})|signs$
  return x

function DifferenceMasks$(VP^A, VN^A, S^A_{before}, VP^B, VN^B, S^B_{before})$
  if $S^B_{before} - S^A_{before} > popcount(VN^B) + popcount(VP^A)$ **then**
    return $M_{A>B} = 0^w, M_{B>A} = 1^w$
  if $S^B_{before} - S^A_{before} = 2w$ **and** $VN^B = 1^w$ **and** $VP^A = 1^w$ **then**
    return $M_{A>B} = 0^w, M_{B>A} = 1^w \ \& \sim(1 \ll (w-1))$
  if $S^B_{before} - S^A_{before} = 0$ **and** $VN^B = 1^w$ **and** $VP^A = 1^w$ **then**
    return $M_{A>B} = 1^w, M_{B>A} = 0^w$
  $D^A \leftarrow (M_{sign} + \text{CPFS}(VN^A, 0) - \text{CPFS}(VN^B, 0)) \wedge M_{sign}$
  $D^B \leftarrow (M_{sign} + \text{CPFS}(VN^B, S^B_{before} - S^A_{before}) - \text{CPFS}(VN^B, 0)) \wedge M_{sign}$
    ▷ D^A and D^B now contain the values $S^A - S^A_{before}$ and $S^B - S^A_{before}$ at each chunk boundary in two's complement
  $M_{smear} \leftarrow ((D^B \ \& \ M_{sign}) \gg (chunksize - 1)) * ((1 \ll (chunksize - 1)) - 1)$ ▷ 1 where D^B needs to be deducted from D^A and 0 elsewhere
  $D \leftarrow$ AddC(DeductC$(D^A, D^B \ \& \sim M_{smear} \ \& \ M_{sign}), \sim D^B \ \& \ M_{smear} + M_{smear} \ \& \ M_{lsb})$ ▷ D now contains the value difference $S^A - S^B$ at each chunk boundary
  for $i \in [0, chunkSize - 1]$ **do** ▷ Calculate the result masks at each $chunkSize$'th bit in parallel
    $D \leftarrow$ AddC$(D, (VP^A \gg i) \ \& \ M_{lsb} + (VN^B \gg i) \ \& \ M_{lsb})$
    $D \leftarrow$ DeductC$(D, (VN^A \gg i) \ \& \ M_{lsb} + (VP^B \gg i) \ \& \ M_{lsb})$
    $M_{D<0} \leftarrow D \ \& \ M_{sign}$
    $M_{D\neq 0} \leftarrow ((D|M_{sign}) - M_{lsb}) \ \& \ M_{sign}$
    $M_{B>A} \leftarrow M_{B>A}|(M_{D<0} \gg (chunkSize - i - 1))$
    $M_{A>B} \leftarrow M_{A>B}|(M_{D\neq 0} \ \& \sim M_{D<0} \gg (chunkSize - i - 1))$
  return $M_{A>B}, M_{B>A}$

function MergeBitvectors$(VP^A, VN^A, S^A_{before}, S^A_{end}, VP^B, VN^B, S^B_{before}, S^B_{end})$
  $M_{A>B}, M_{B>A} \leftarrow$ DifferenceMasks$(VP^A, VN^A, S^A_{before}, VP^B, VN^B, S^B_{before})$
  $M_p \leftarrow (M_{A>B}|((M_{B>A}|M_{A>B}) - (M_{A>B} \ll 1))) \ \& \sim M_{B>A}$
  $VN^A_{reduced} \leftarrow VN^A \ \& \sim(M_{B>A} \ \& \ (M_{A<B} \ll 1))$
  $VN^B_{reduced} \leftarrow VN^B \ \& \sim(M_{A>B} \ \& \ (M_{B<A} \ll 1))$
  $VP^{out} \leftarrow VP^B \ \& \ M_p + VP^A \ \& \sim M_p$
  $VN^{out} \leftarrow VN^B_{reduced} \ \& \ M_p + VN^A_{reduced} \ \& \sim M_p$
  return $S^{out}_{before} = \min(S^A_{before}, S^B_{before}), S^{out}_{end} = \min(S^A_{end}, S^B_{end}), VP^{out}, VN^{out}$
